# Skeletal Calcium/Phosphorus Ratio Measuring Techniques and Results. I. Microscopy and Microtomography

**DOI:** 10.1100/tsw.2004.200

**Published:** 2004-11-30

**Authors:** Vassilios Kyriazis, Margaret Tzaphlidou

**Affiliations:** ^1^ Department of Materials Science and Technology, University of Ioannina, 45110 Ioannina, Greece; ^2^ Medical Physics Laboratory, Medical School, University of Ioannina, 45110 Ioannina, Greece

**Keywords:** transmission electron microscopy, micro-computed tomography, skeletal Ca/P ratio

## Abstract

An approach to the problem of bone disorders is the measurement of the skeleton's static and dynamic strength, an estimate of which is bone mineral density. A decrease in the latter may be due to a decrease in either Ca or P, or to dissimilar decreases in both. Consequently, the determination of the Ca/P ratio may provide a sensitive measure of bone mineral changes and may add to our understanding of the changes occurring in bone diseases. This paper reviews techniques such as transmission electron microscopy (TEM) and micro-computed tomography (μu-CT), which have been developed for the *in vitro* assessment of the Ca,P content and the skeletal Ca/P ratio. Their main aspects are presented, as much as results regarding the referred values. The presentation of other *in vitro* or *in vivo* techniques, such as instrumental neutron activation analysis (INAA) or X-ray absorptiometry accordingly, would be the issue of another article. The authors argue that the choice of the best technique relies on its cost ad ease of applicability, its reliability, and precision.

### INTRODUCTION

For over 150 years, bone mineral has been known by chemical analyses to contain calcium (Ca) and phosphorus (P). In 1926, the similarity between the X-ray diffraction pattern of bone powder and the pattern observed from the basic calcium phosphate mineral, hydroxyapatite, was first observed[1].

Mineralized phase of the bone is so-to-say composed of hydroxyapatite [$Ca_{10}(PO_4)_6(OH)_2$] in a crystalline form, structurally specific for bone. Its crystallinity varies, however, depending on the stage in the mineralization process and age[2]. Bone also contains collagen (mainly type I), marrow, fat, noncollagenous proteins, and water[3].

The theoretical fraction of Ca in hydroxyapatite is 40.3% and P 18.4%[4]. However, the Ca and P content of hydroxyapatite in human bone may not correspond to these values, as shown in studies employing chemical or instrumental neutron activation analysis (INAA), where the values vary between 18.5–62% for Ca and 8.7–27% for P[4,5]. Since bone is a biomaterial that is structurally adapted to different functions and loading situations in adults, mainly as cortical and trabecular bone, its exact composition may vary. The main influencing factors for this variation are the bone type and location in the human body[6], but also some alterations that are known to occur in bone metabolic diseases.

The incidence of bone diseases such as osteoporosis increases with advancing age and the fractures are usually the result of relatively minor trauma. Such a situation represents a significant medical and social problem in terms of treatment and rehabilitation. Therefore, a reliable early diagnosis of a bone disorder is needed[7]. A significant approach to this problem is the measurement of the static and dynamic strength of the skeleton. The most common way to estimate this bone strength is based on the assessment of bone mineral density, without considering the role played by the different chemical components that are present. However, recent reports[8] suggest that the accuracy of the data to be obtained by this method is insufficient for such a purpose. A decrease in bone density may be due to a decrease in either Ca or P, or to dissimilar decreases in both. Consequently, the determination of the Ca/P ratio may provide a sensitive measure of bone mineral changes and may add to our understanding of the changes occurring in bone diseases[9]. A new method has recently been demonstrated for assessing the skeletal Ca/P ratio *in vivo*[10,11]. To provide a fuller understanding of these results, *in vitro* studies have been designed too. Under these conditions, this article reviews some of the up-to-date techniques that have been developed for the determination of the skeletal Ca/P ratio and presents according results.

### TRANSMISSION ELECTRON MICROSCOPY (TEM)

#### Basic Principles of Function

This technique is used for the Ca/P ratio measurements, not only in cases of normal subjects, but also in cases of bone diseases (e.g., Osteogenesis Imperfecta [OI]). To the best of our knowledge, TEM has been used for bone studies from the 1980s[1,12]. For the application of the method, biopsy bone specimens follow a typical preparation procedure for the transmission electron microscope.

An alternative is the method of cryoprocessing, where minute pieces (about 0.5 $mm^3$) are attached onto the groove of a silver cryopin on a film of 2.3 *M* sucrose in 0.1 *M* sodium cacodylate buffer[12]. With the use of this technique, artifactual changes in the mineral composition of bone that can occur in aqueous media can be avoided; thus, better opportunities for accurate and precise electron probe microanalysis are offered. The pin, with the bone attached to it, is immediately frozen by immersion in liquid nitrogen and transferred to the cryochamber of the ultramicrotome for sectioning.

In both alternatives, specimens are viewed uncontrasted in a transmission electron microscope and further analyzed with specific microanalysis systems (e.g., in [12]). Specimens are analyzed for several seconds at a most appropriate accelerating voltage that gives a good peak-to-background ratio. A number of areas are counted randomly over the dense mineral of the examined bone of each specimen. The “background” or continuum radiation is automatically subtracted by the microanalysis program.

#### Outlines and Limitations

TEM implies a number of constraints and limitations in its use; one such is the specimen preparation, which is time consuming and requires skill and attention by its user. Moreover, only 2-D pictures of the studied samples are obtained and these require the development of specific software for further processing.

All the same, unlike the technique's significant cost of purchase and maintenance, it is easily accessible for research applications in several laboratories and medical units worldwide; this applicability is targeted exclusively into fundamental research and the status of the studied samples is *in vitro*. The latter presents certain inferiority, since according to the writers' view a technique should target in measurements *in vivo*.

### Measurements

Table 1 presents the results derived from the literature. It is shown[1] that the Ca/P ratio for the normal bone via TEM is 1.631 (for the synthetic hydroxyapatite the according value is 1.602). There is a systematic difference in the Ca/P ratio between normal and OI of various types' bone, which for the latter is 1.488. Furthermore, no age- or sex-related deviations are observed in the OI bone samples.

**TABLE 1**
**Results for the Ca/P Ratio on Human Bones Derived from the Literature with the Use of TEM on Cryosections or Resin Sections**

| Method | Disease/Bone Type | Ca/P | Ref. |
|---|---|---|---|
| Resin sections with TEM | Normal/— | 1.631 | [1] |
| | OI of various types/— | 1.488 | |
| Cryosections with TEM | Normal/femoral fetal | 1.68±0.03 | [12] |
| Resin sections with TEM | Normal/femoral fetal | 1.69±0.03 | |
| Cryosections with TEM | OI type II/femoral fetal | 1.49±0.06 | |
| Resin sections with TEM | OI type II/femoral fetal | 1.55±0.05 | |

Sarathchandra et al.[12] prove that the Ca/P ratio of OI type II in resin sections (classical TEM technique) demonstrates lower values (1.55) than the normal ones (1.69). The results of the cryosections are even more emphatic: for the OI type II the computed Ca/P ratio is 1.49. The same authors argue that these changes may be associated with abnormal collagen; this, however, is a proven fact, observed through the use of inflammation mediated osteoporosis (IMO)[13].

## MICRO-COMPUTED TOMOGRAPHY (μ-CT)

### Basic Principles of Function

Conventional clinical imaging techniques do not fulfill requirements that are satisfied by a fully 3-D, nondestructive imaging system in bone measurements. Since 2-D analysis tools only give an estimate of the 3-D bone structural properties, and also high spatial resolution is required for such measurements, specific devices have been proposed for the investigation of bone *in vitro*. For example, magnetic resonance imaging (MRI) has recently been used in bone research offering a spatial resolution around 100 $\mu m^3$. Computed microtomography (μ-CT), which is similar to conventional computed tomography (CT) widely used in medicine, but with higher spatial resolution, is particularly well suited for bone structure studies[14]. Different X-ray μ-CT systems using laboratory X-ray sources have been developed for bone structure studies, the first of which was introduced in the late 1980s[15]. The basic assumption of this technique is that the μ-CT image is a map of the linear attenuation coefficient of an X-ray beam passing through a bone sample at different viewing angles; in this way, measurements of the linear attenuation coefficient value are taken and this attenuation is related to the amount of the mineral content, which in its term is related to the differences in gray levels in reconstructed images[16]. Each element of the projection is a line integral of the linear attenuation coefficient value in the sample along the X-ray beam path. A map of this coefficient can be recovered from these line integrals using suitable reconstruction algorithms. Different X-ray microtomography systems using laboratory X-ray sources have been developed in this way for bone structure studies[15,17,18,19,20,21].

Synchrotron radiation (SR) provides outstanding properties for μ-CT and makes it possible to reach a spatial resolution down to the micron level. The use of a parallel beam in SR μ-CT allows exact reconstruction of the image, which is not possible for cone beam geometries used in most μ-CT. In this case, the standard X-ray beam on conventional μ-CT devices is replaced with a high photon flux monochromatic X-ray beam extracted from synchrotron beam; so to say, 3-D images with a very high spatial resolution, high contrast, and signal-to-noise ratio are provided via appropriate image reconstruction software. The image from SR shows sharp outlines of bone structures and differences in gray levels, while blurring, distortions, and beam-hardening artifacts are observed using standard X-ray tube μ-CT. Thus, SR and conventional μ-CT present significant differences in the quantitative reconstruction of the linear attenuation coefficient.

The monochromaticity of the beam is a key point, since this condition is a perfect assumption for tomographic reconstruction. Energy tunability is also useful to identify different elements in a sample using dual energy techniques. The high photon flux available from synchrotron X-ray sources and the small angular source size leading to negligible geometrical blur, allow us to achieve images with high spatial resolution and a high signal-to-noise ratio.

The use of SR as a source for high-resolution μ-CT of small samples was first suggested by Grodzins[22] and has been implemented at several SR facilities around the world. Like in CT, different setups have been proposed for data acquisition. First-generation scanning systems use a pencil beam and a single detector[23,24]. The sample is successively rotated and translated through the beam. This technique allows reaching a spatial resolution of the order of 1 μm, since the pixel size is determined by the beam size, but is rather time consuming, particularly to achieve 3-D measurements. Another solution consists in using a linear detector, for example a photodiode array, which avoids translating the sample through the beam[25,26,27]. The fastest technique to obtain 3-D images is to acquire data for all slices simultaneously using a 2-D detector. This is known as truly 3-D tomography; the whole volume may be reconstructed from 2-D projections acquired at different angles of view[28,29,30]. In the case of SR, the divergence of the beam is very small and does not provide any direct magnification. The spatial resolution in the image for this nearly parallel beam is thus determined by the spatial resolution of the detector. The use of an asymmetric channel-cut crystal[31] has been applied to bone structure studies[32,33].

### Outlines and Limitations

SR μ-CT implies a number of constraints and a higher expense, compared to μ-CT systems based on laboratory X-ray sources. SR sources are less accessible than conventional X-ray sources. However, SR provides characteristics that cannot be found elsewhere, in particular monochromaticity and high photon flux, which is absolutely necessary to reach a spatial resolution as high as 1 μm. These characteristics fully justify the use of SR, in spite of the constraints associated to it.

The limited access to SR makes it difficult to perform measurements on a very large series of samples. This point is a severe limitation in biological sciences, which require the study of a large number of specimens to be able to draw statistical conclusions. Nevertheless, the progress made in terms of scan time optimization can reduce the acquisition of a complete dataset to a limited time, which allows the imaging of a large number of samples.

Another limitation is the limited size of the examined samples. Since the number of pixels in the detector can be fixed, the higher the spatial resolution, the smaller the field of view.

Due to dose considerations, this high-resolution imaging technique is not applicable *in vivo* to human imaging, unless on biopsies. Due to the constraints described above, μ-CT is perfectly adapted to fundamental research and can be also used as a calibration technique for clinical methods with coarser spatial resolution like conventional scanners of MRI.

### Measurements

μ-CT has been applied in animal bone samples for the assessment of the Ca/P ratio in different bone sites. The initial scans were performed *in vitro*. Relatively recently, some *in vivo* scans were acquired in rats, mainly for determination of bone mineral mass or density[34], but with relatively low spatial resolution. All the same, μ-CT with high spatial resolution has been performed *in vivo* in invertebrate animals[35], namely snails, which were chosen because they are known to grow fast, their shells develop simultaneously with their body, and can be easily mounted on the μ-CT system for testing. Although the results via 3-D reconstruction cross-section images do not refer to a Ca/P ratio, they indicate that these animals resisted the repetitive exposure to X-rays very well, grew well during the experiments, and were able to regenerate their shells, where these were artificially damaged. However, due to the low soft tissue contrast, their body could not be clearly detected and visualized. Moreover, with the help of μ-CT systems, results regarding the morphologic (bone volume to total tissue volume ratio, bone surface to bone volume ratio, trabecular thickness, number, and separation), as much as the topologic parameters (connectivity and connectivity density) of the femoral cortical and trabecular bones in rats are computed *in vitro* and assessed[36].

A system based on the combination of X-ray projection microscopy with a tomographical reconstruction technique has been developed for measuring bone Ca content using high-resolution desktop μ-CT by Postnov et al.[37]. Bones from adult, normal mice were isolated from normal parts of the skeleton (ribs, femur, and vertebral column), dried and scanned for the determination of the Ca concentration. In this system, an air-cooled point X-ray source (focal point size ~8 μm in diameter, maximum voltage 80 kV) was used to illuminate the object with a divergent beam. Magnified shadow pictures were detected by a 2-D CCD camera. Cross-sections were reconstructed with a specifically designed algorithm. All samples and phantoms were scanned under identical conditions. For μ-CT analysis, bone was considered as a crystalline fraction and an organic matrix. The contribution to X-ray absorption of each component in bone was calculated and measured. As indicated by the results, specific X-ray absorption occurred in all elements. The results were based on image analysis of the local gray values in the reconstructed gray sections and are presented in Table 2.

As it is known that the mechanical strength of the bone depends first of all on the condition of the cortical bone[38], normal and osteoporotic rabbit cortical bone from the femoral neck and tibia regions was analyzed by Tzaphlidou et al.[38]. Sample data were collected at 20 and 28 keV. These two energies were chosen as the best compromise that provided the greatest energy separation, but that also took into account the competing processes of photon attenuation in the sample and photon intensity at the source. The results derived are viewed in Table 2. These results show a significant difference between osteoporotics and age-matched normals at both energies. Differences between different bone sites from the same animal are not significant, while those between the same bone sites from different animals are highly significant[38]. Differences between estimates made at 20 and 28 keV are small, without any statistical significance.

However, an important aspect that is unique to the measurements made by Tzaphlidou et al.[38] is the ability to map the spatial distribution of the Ca/P ratio. This has been investigated by using contour plots for selected slices where the local gradients exhibited in the contour plots are a measure of the changing Ca/P ratio and hence can be used to study the changing distribution[38]. The results confirm that a statistically significant decrease is observed in rabbit tibia Ca/P ratio for osteoporotic samples, suggesting that there is a relationship between bone loss and a lowered Ca/P ratio. Also, it is seen that a sample that is judged to be osteoporotic from the bulk estimate of the Ca/P ratio may show regions of osteoporotic behavior and regions of normal behavior. Further work is required to confirm and understand these observations and their full interpretation is still ongoing.

**TABLE 2**
**Results for the Ca Content and the Ca/P Ratio Derived from the Literature with the Use of μ-CT on Animal Bone Samples**

| Method | Disease/Bone Type | Ca | Ca/P (20 keV) | Ca/P (28 keV) | Ref. |
|---|---|---|---|---|---|
| μCT | —/Mouse femur 1 | 0.25 | — | — | [37] |
| | —/Mouse femur 2 | 0.24 | — | — | |
| | —/Mouse femur 3 | 0.25 | — | — | |
| | —/Mouse ribs | 0.19 | — | — | |
| | —/Mouse vertebral column | 0.19 | — | — | |
| | —/Newts control | 0.154 ± 0.005 | — | — | |
| | —/Newts space | 0.139 ± 0.011 | — | — | |
| SR μCT | —/Femoral rabbit 1 | — | 1.55 ± 0.18 | 1.54 ± 0.37 | [38] |
| | —/Femoral rabbit 2 | — | 1.22 ± 0.19 | 1.14 ± 0.26 | |
| | —/Tibia rabbit 1 | — | 1.88 ± 0.06 | 1.84 ± 0.31 | |
| | —/Tibia rabbit 2 | — | 1.79 ± 0.05 | 1.97 ± 0.05 | |
| | Osteoporosis/tibia rabbit 1 | — | 1.45 ± 0.04 | 1.67 ± 0.18 | |
| | Osteoporosis/tibia rabbit 2 | — | 1.66 ± 0.06 | 1.75 ± 0.13 | |
| | —/Tibia lamb 1 | — | 1.01 ± 0.04 | 0.96 ± 0.05 | |
| | —/Tibia lamb 2 | — | 1.19 ± 0.04 | 1.05 ± 0.06 | |
| | —/Tibia sheep 1 | — | 1.28 ± 0.04 | 1.17 ± 0.07 | |
| | —/Tibia sheep 2 | — | 1.55 ± 0.05 | 1.65 ± 0.04 | |

Due to the importance of the condition of the cortical bone — as already mentioned — bone samples of lambs, rabbits, and rats have been assessed for their Ca/P ratio using SR μ-CT (unpublished results). Mean values for Ca/P ratios between different bone sites and different animals are highly significant, demonstrating a dependence on lifestyle and bone use of these species.

### DISCUSSION

This article reviews techniques such as TEM and μ-CT, which have been developed for the determination of the skeletal Ca and P content, as much as of the value of their ratio in various bone types. The review of other techniques and the results derived by their use in the determination of the same values, such as INAA or X-ray absorptiometry, should be the aim of another article. Both techniques presented here are oriented to perform *in vitro* measurements. SR μ-CT is less accessible than TEM, but has greater resolution even than that of conventional μ-CT; this resolution reaches the micron level. Unlike via μ-CT, a large number of samples can be processed with the use of TEM; however, via the latter method, only 2-D images are derived and special software has to be developed for image processing.

However, in the authors' view, in every case, the choice of the most appropriate technique should rely on the cost of purchase or construction of the necessitated equipment, as much as on the cost of maintenance. The resolution and the accuracy of the measured values should also be a critical choice factor for a technique. Moreover, the accuracy of measurements and the *in vivo* or *in vitro* status of the studied material should be an important factor. According to the authors' views, a laboratory should begin with the simplest in construction, use, and maintenance technique and move forward to more sophisticated and costly techniques.

Because bone with an origin of a disease has histological differences from the normal one, which are present mainly as an increase of the bone nonmineralized fraction[9], one can use techniques such as TEM and μ-CT to detect deviations from according normal values. In this way, the *in vitro* results presented in Tables 1 and 2 show that the Ca/P bone ratio in cases of diseases such as OI or osteoporosis is systematically lower than that derived from normal bone samples, indicating that it can be used as an index of bone quality. Further work on bone samples by TEM or/and μ-CT can create a solid database that can be useful in the assessment of the accuracy of *in vivo* Ca/P ratio techniques, as much as of bone diseases.

In conclusion, as has been reported[39,40,41], the Ca/P ratio may provide greater reliability for diagnosis of bone disorders and hence the described techniques may become valuable to be used during bone therapeutic and diagnostic trials.
